# Clinicopathological features of dysembryoplastic neuroepithelial tumor: a case series

**DOI:** 10.1186/s13256-023-04062-1

**Published:** 2023-08-01

**Authors:** Shabina Rahim, Nasir Ud Din, Jamshid Abdul-Ghafar, Qurratulain Chundriger, Poonum Khan, Zubair Ahmad

**Affiliations:** 1 Department of Pathology and Laboratory Medicine, Aga Khan University Hospital, Karachi, Pakistan; 2 Department of Pathology and Clinical Laboratory, French Medical Institute for Mothers and Children (FMIC), Kabul, Afghanistan; 3 Department of Radiology, Aga Khan University Hospital, Karachi, Pakistan

**Keywords:** Dysembryoplastic neuroepithelial tumors, Epilepsy, Seizure

## Abstract

**Background:**

Dysembryoplastic neuroepithelial tumors are rare benign supratentotrial epilepsy-associated glioneuronal tumors of children and young adults. Patients have a long history of seizures. Proper surgical resection achieves long term seizure control. We describe the clinicopathological features of dysembryoplastic neuroepithelial tumor cases reported in our practice and review the published literature.

**Methods:**

All cases of Pakistani ethnicity were diagnosed between 2015 and 2021 were included. Slides were reviewed and clinicopathological features were recorded. Follow-up was obtained. Extensive literature review was conducted.

**Results:**

Fourteen cases were reported. There were 12 males and 2 females. Age range was 9–45 years (mean 19 years). Majority were located in the temporal and frontal lobes. Duration of seizures prior to resection ranged from 2 months to 9 years with mean and median duration of 3.2 and 3 years, respectively. Histologically, all cases demonstrated a multinodular pattern, specific glioneuronal component, and floating neurons. Simple and complex forms comprised seven cases each. No significant nuclear atypia, mitotic activity, or necrosis was seen. Ki-67 proliferative index was very low. Cortical dysplasia was noted in adjacent glial tissue in four cases. Follow-up ranged from 20 to 94 months. Seizures continued following resection in all but one case but were reduced in frequency and intensity. In one case, seizures stopped completely following surgery.

**Conclusion:**

Clinicopathological features were similar to those in published literature. However, a marked male predominance was noted in our series. Seizures continued following resection in all but one case but were reduced in frequency and intensity. This series will help raise awareness among clinicians and pathologists in our part of the world about this seizure-associated tumor of children and young adults.

## Introduction

In developing countries such as Pakistan, many neurosurgeons, neurooncologists, and neuropathologists are unaware of these epilepsy-associated tumors. Absence of and lack of access to proper pediatric and adult epilepsy surgery programs and centers results in delay in the detection and proper treatment of these tumors. Dysembryoplastic neuroepithelial tumors (DNTs) are rare benign mixed glioneuronal epilepsy-associated tumors occurring in children and young adults [1]. DNTs comprise 1.2% and 0.2% of all central nervous system (CNS) tumors in patients under 20 and above 20 years of age, respectively [2]. DNTs are mostly located in the supratentorial cortex. Over 67% are located in the temporal lobe with preferential involvement of mesial structures. The frontal lobe is the second most common location [3]. Patients typically present with drug-resistant focal epilepsy, usually without any neurological deficits. Seizures may become generalized in the long term. In a study of epilepsy-associated tumors, DNTs comprised 23.4% and 17.8% of long-term epilepsy-associated tumors in children and adults, respectively [4]. The spectrum of long-term low-grade epilepsy-associated brain tumors is expanding rapidly and, in addition to DNTs, includes diffuse astrocytoma MYB or MYBL1-altered polymorphous low-grade neuroepithelial tumor of the young (PLNTY), diffuse low-grade glioma, MAPK pathway-altered tumor, and so on [5]. In almost 90% of DNTs, the first seizure occurs before 20 years of age. Age of seizure onset ranges from 3 weeks to 38 years, mean age at seizure onset is 15 years [6]. Mean age at epilepsy surgery and histopathological diagnosis is 25.8 years [7]. Mean duration of seizures prior to surgical intervention is 10.8 years [1]. Magnetic resonance imaging (MRI) is the best modality to detect these lesions in children and young adults who present with focal epilepsy so that early surgical interventions can be performed to achieve long term seizure control [5, 8–10].

On imaging, these lesions usually encompass the thickness of cerebral cortex. No mass effect or significant tumoral edema are seen, which are important criteria in differentiating DNTs from diffuse gliomas [1]. On MRI, most tumors present as T2 hyperintense single or multiple pseudocysts [3]. Minimal or no enhancement is noted in these lesions, which is also an important distinguishing feature from ganglioglioma, which shows strong enhancement [53]. In fluid-attenuated inversion recovery (FLAIR) images these lesions appear as mixed signal intensity with bright rim sign, which is a specific sign [54]. In one third of cases, calcifications may be seen in DNT, which are best seen on computed tomography (CT).

DNTs usually have a multinodular architecture. Histologically, DNTs demonstrate a specific glioneuronal element characterized by columns made up of bundles of axons oriented perpendicular to the cortical surface and lined by oligodendrocyte-like cells embedded in a mucoid matrix. Interspersed floating neurons are characteristic. Simple and complex forms are recognized. In the former, only the unique glioneuronal element is seen. In the latter, glial nodules are seen together with the unique glioneuronal element and give DNTs their characteristic multinodular architecture. Glial nodules may resemble diffuse glioma (astrocytoma or oligodendroglioma) or mimic pilocytic astrocytoma [1]. Ishizawa *et al.* reported a case that showed a pleomorphic xanthoastrocytoma-like component [11]. Some DNTs do not show either the specific glioneuronal element or multinodularity. In such cases, distinguishing them from diffuse gliomas is extremely difficult. DNTs correspond histologically to World Health Organization (WHO) grade 1. Ki-67 proliferative indices are very low (< 1%). Long-term prognosis following epilepsy surgery is excellent and recurrence or progression are exceptional [12–14].

In developing countries, there is less awareness among pathologists and clinicians regarding epilepsy-associated tumors. Limited expertise in epilepsy associated tumors often results in delays in tumor detection and proper treatment, which impedes the aim of achieving long-term seizure control. The aim of this study is to describe the clinicopathological features of DNTs diagnosed in our practice and to present a detailed review of the published literature. We hope that our study will raise awareness about these important epilepsy-associated neoplasms among neurosurgeons, oncologists, and neuropathologists in our region and contribute to better and timely care of children and young adults suffering from long-term epilepsy secondary to these relatively rare tumors.

## Material and methods

The surgical pathology files of a tertiary care center were searched for cases of DNTs. Cases diagnosed between January 2015 and December 2021 were included. Hematoxylin and eosin (H&E) and immunohistochemical (IHC) slides of all cases were retrieved and reviewed by the two senior authors (ZA and NU). Clinicopathological features of all cases were described and follow-up was obtained. Extensive literature review was conducted. Ethical approval was obtained from the institution’s ethical review committee (ERC).

## Results

**Fig. 1:**
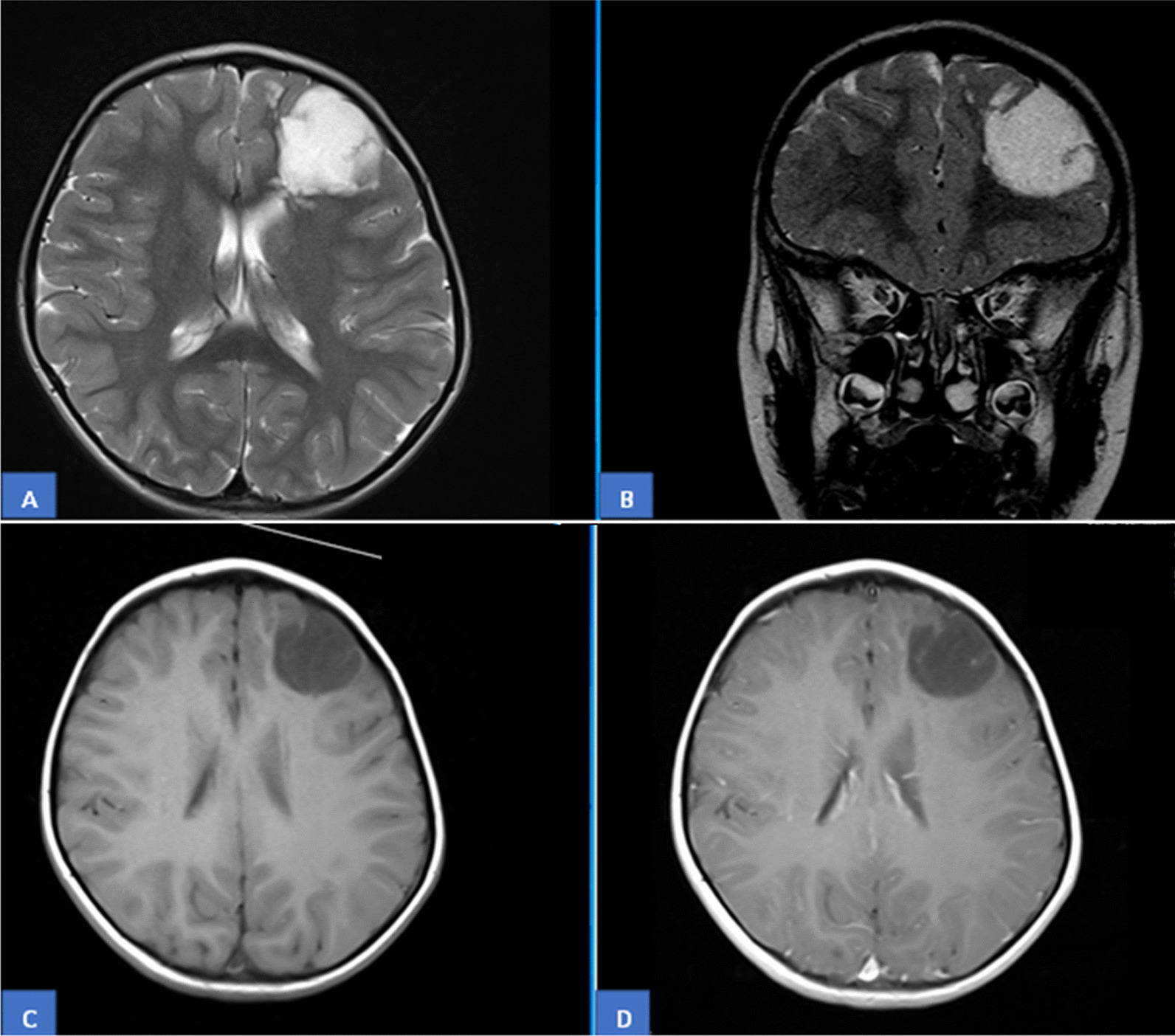
MRI images: **A**, **B** A well-defined lobulated T2W cystic cortical and subcortical left frontal lobe lesion is identified. No enhancement is seen on post contrast axial and sagittal images. **C**, **D** Peripheral rim sign on FLAIR images. No signal dropout seen on SW1 sequence. Displacement of white matter tracts seen, suggestive of low-grade less aggressive lesion

Fourteen cases were reported during the study period. Seven cases each were received from our own center and from hospitals/centers in other cities and towns of the country. Twelve patients (85.7%) were males and two (14.3%) were females. Male to female ratio was 6:1. Ages ranged from 9 to 45 years with mean and median age of 19 and 15 years, respectively. Eight patients (57.1%) were 15 years or younger, while 6 (42.9%) were 10 years or younger in age. Duration of seizures ranged from 2 months to 9 years. Mean and median duration was 3.2 years and 3 years, respectively. Headache and vomiting were the most common presenting features. One patient had a history of urinary incontinence while sleeping. Temporal lobe was the most common location (nine cases, 64.3%), followed by the frontal lobe (four cases, 28.6%). One case was parasagittal in location. Out of the nine temporal DNTs, three were temporoparietal while one involved the hippocampus as well. Of the four frontal DNTs, one case each involved the parietal lobe and parasagittal cortex as well. Seven cases each were right- and left-sided in location. All six cases for which radiology was available showed wedge-shaped T2 hyperintense and T1 hypointense lesions with classical soap bubble appearance (Fig. 1A, B). The base of these lesions was oriented towards cortex, and the apex was pointing towards ventricles. No perilesional edema or mass effect was noted in these lesions. None of the lesions showed diffusion restriction or post-contrast enhancement (Fig. 1C, D). However, peripheral rim sign on FLAIR images was seen in one case. No signal dropout was noted on susceptibility weighted imaging (SWI) sequence to suggest tumoral bleed or calcifications. Diffusion tensor imaging (DTI) was performed in two patients, which showed displacement of white mater tracts rather than disruption of fibers, suggesting a lower grade less aggressive neoplasm.

**Fig. 2:**
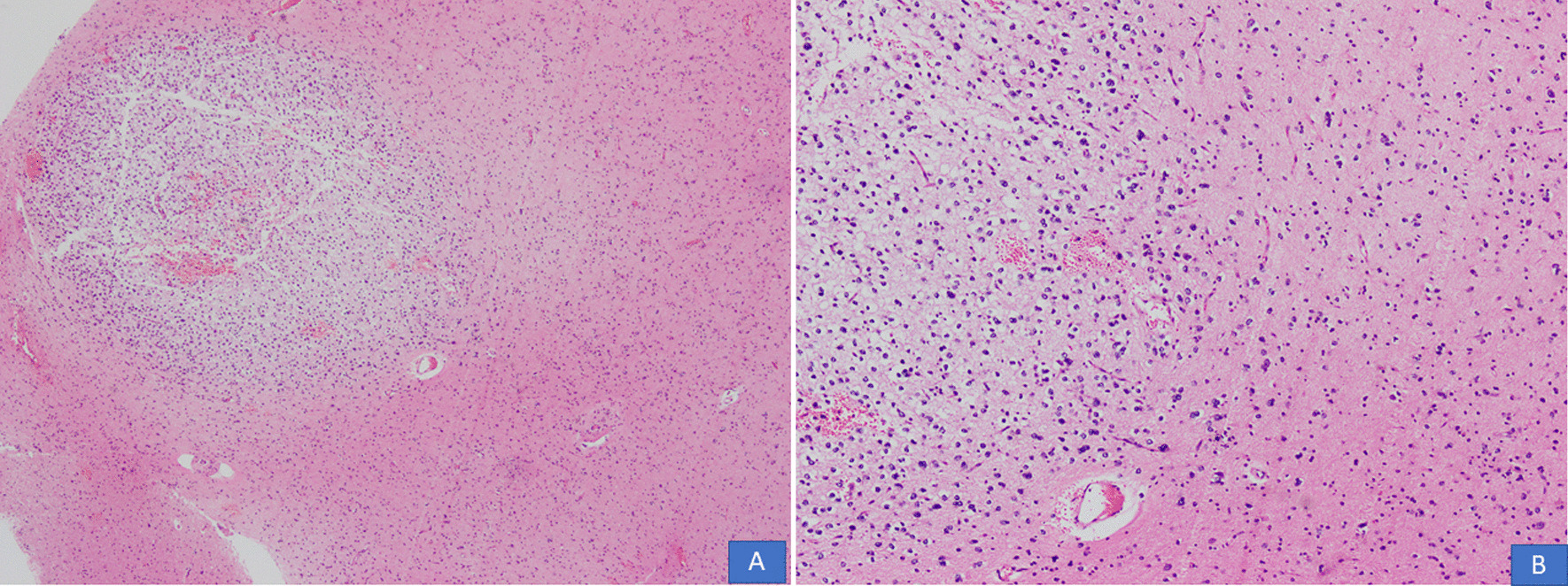
**A**, **B** DNT demonstrating a multinodular growth pattern (H&E, ×100 and ×200 magnifications)

**Fig. 3:**
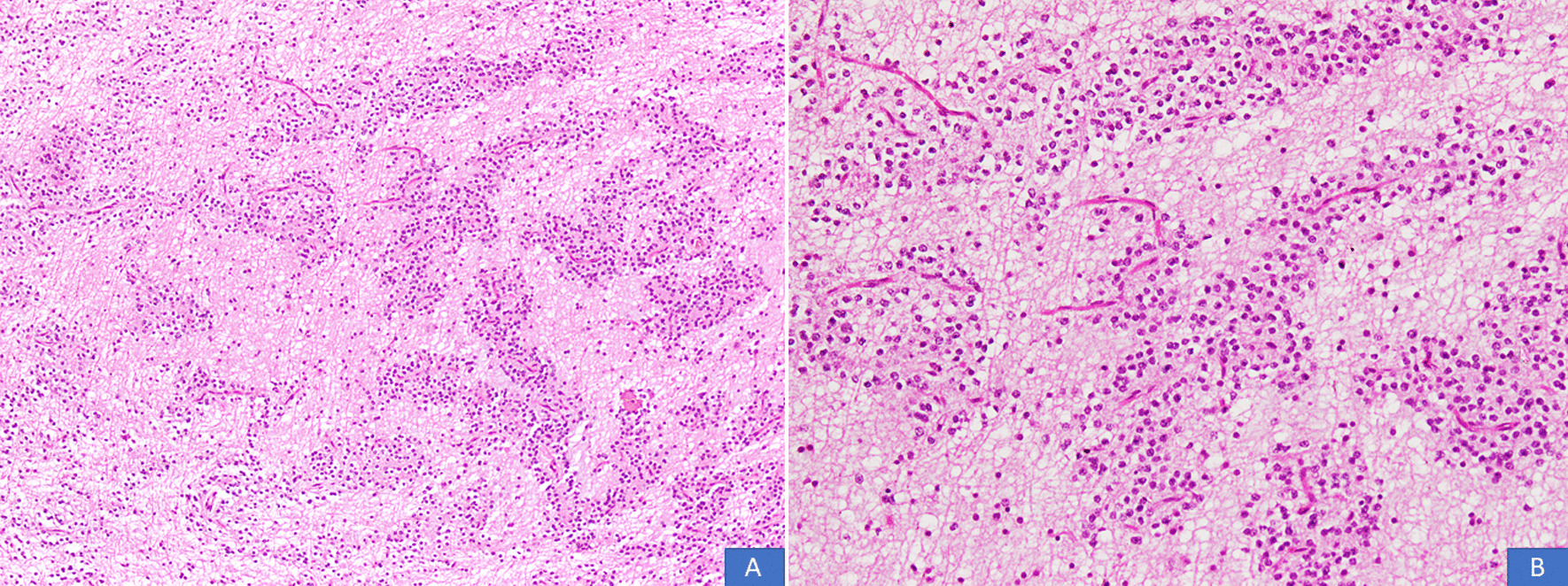
**A**, **B** Columns of oligodendrocyte-like cells embedded in a mucoid matrix (H&E, ×100 and ×200 magnifications)

**Fig. 4:**
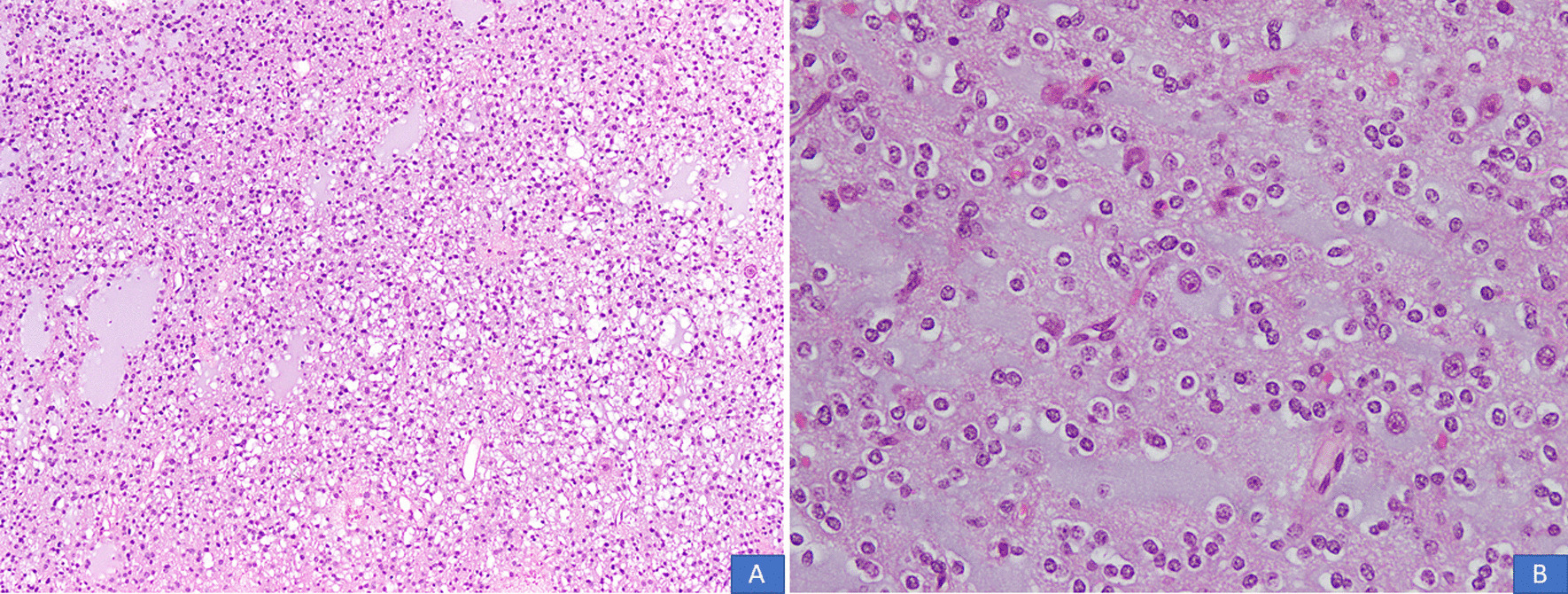
**A**, **B** Oligodendroglioma-like areas and mucin filled cystic areas (H&E, ×100 and ×200 magnifications)

**Fig. 5:**
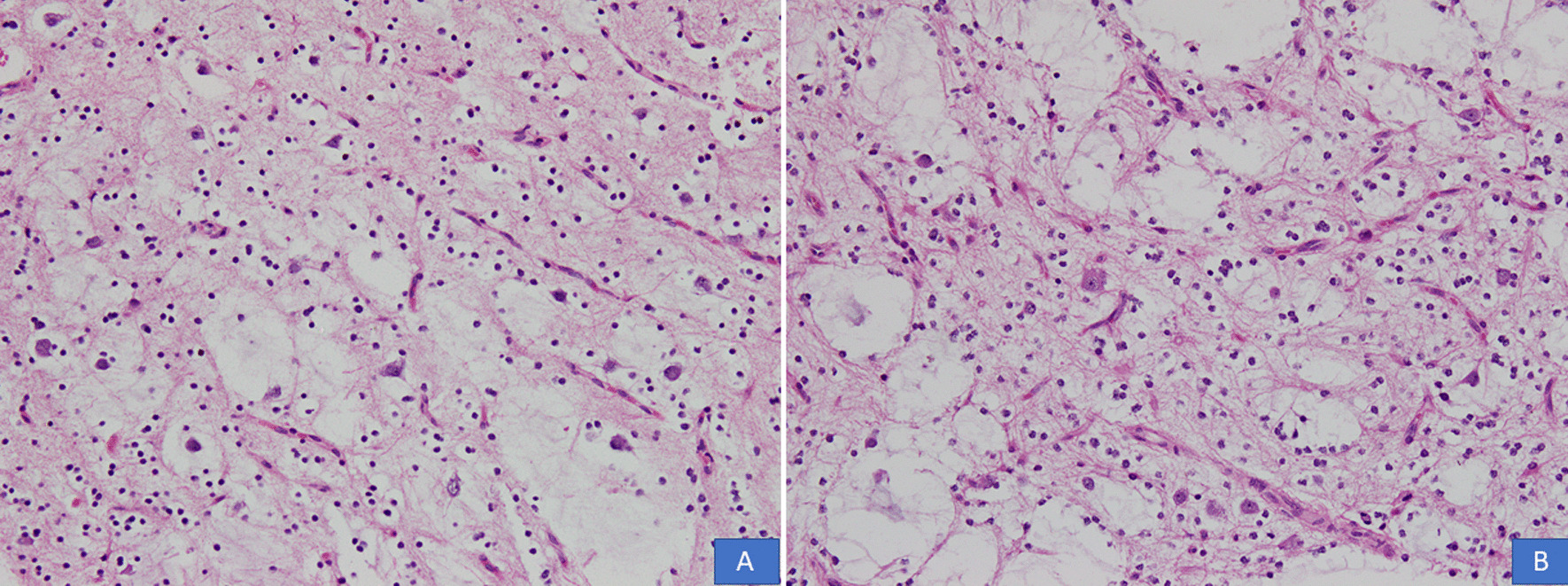
**A**, **B** Floating neurons in the specific glioneuronal component (H&E, ×100 and ×200 magnifications)

**Fig. 6:**
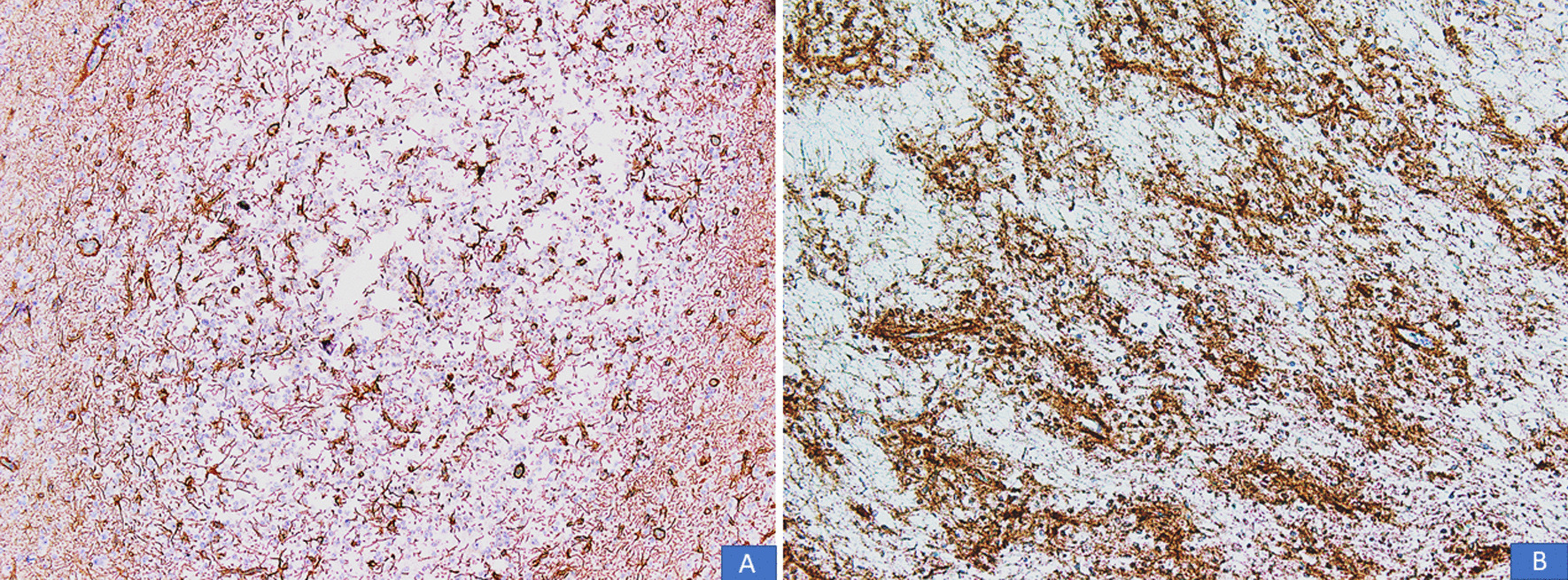
**A** Immunoreactivity for GFAP seen in scattered stellate astrocytes within the specific glioneuronal elements. Oligodendrocyte-like cells are absent. **B** Immunoreactivity for synaptophysin seen in neuropil-like matrix components, especially around blood vessels

All cases were received in the form of multiple pieces ranging from 1.0 cm to 11 cm in aggregate. Mean and median size was 4.6 cm and 4 cm, respectively. On histological examination, all cases demonstrated a multinodular growth pattern (Fig. 2A, B), and the specific glioneuronal element composed of columns oriented perpendicular to the cortical surface and made up of bundles of axons lined by oligodendrocyte like cells embedded in a mucoid matrix (Fig. 3A, B). Oligodendroglioma-like areas, mucin filled cystic foci, and floating neurons were seen in all 14 cases (Figs. 4 and 5A, B). Seven cases each (50% each) corresponded to the simple and complex forms. In the latter, histological features corresponded to diffuse glioma, WHO grade 2, in five cases and pilocytic astrocytoma in two cases. Cortical dysplasia was seen in the glial tissue adjacent to the tumor in four cases (28.6%). None of the cases demonstrated significant nuclear atypia, mitotic activity or necrosis. Tumors appeared circumscribed in all cases. Microcalcification was seen in three cases (21.4%). Glial fibrillary acidic protein (GFAP) was negative in the oligodendrocyte-like cells in all cases but was positive in the scattered stellate astrocytes within the specific glioneuronal component. GFAP was positive in the glial areas in complex cases. Synaptophysin was expressed in the floating neurons (Fig. 6A, B). The Ki-67 proliferative index was < 2% in the simple forms and 3%–5% in the two complex cases and was higher in the glial nodules. Follow-up was available in 12 cases and ranged from 20 to 94 months. Median follow-up period was 30 months. Two patients died within days of surgery from acute postsurgical complications. Seizures persisted following surgery in 9 patients but were reduced in frequency and severity. All were started on tablet valproic acid (Epival) 500 mg twice daily, and then the dose was adjusted accordingly after checking valproate levels. In one patient, surgery resulted in complete cessation of seizures and patient is alive and well without needing any antiepileptic medication. At the time of last follow-up, none of the patients had developed recurrence (Table 1).

**Table 1:** Clinical, epidemiological, and follow-up details (*n* = 12)

Study no.	Year of resection	Age at resection (years)	Sex	Location	Alive	Duration of seizures before resection (years)	Seizures postresection	Antiepileptic drugs post resection	Length of follow-up months
1	2014	15	Male	Left temporoparietal	Yes	3	Not controlled	Yes	94
2	2016	10	Male	Right temporoparietal	Yes	Not known	Not controlled	Yes	66
3	2017	9	Male	Right frontal	Yes	1	Not controlled	Yes	52
4	2018	9	Male	Left frontal	Yes	Not known	Not controlled	Yes	46
5	2018	25	Male	Frontoparietal	No**	6	N.A.	N.A.	N.A.
6	2018	12	Male	Left temporal	Yes	Not known	Not controlled	Yes	39
7	2019	10	Male	Parasagittal	Yes	2	Controlled	No	35
8	2019	30	Male	Right temporal	Yes	9	Not controlled	Yes	30
9	2019	32	Male	Right temporal	Yes	1	Not controlled	Yes	28
10	2019	8	Male	Left temporal	Yes	4	Not controlled	Yes	25
11	2020	12	Female	Right frontal	Yes	2 Months	Not controlled	Yes	20
12	2020	30	Female	Right temporoparietal	No**	Not known	N.A.	N.A.	N.A.

N.A., not applicable

^**^Died within days following resection from postsurgical complications

The total sample size comprises 14 young patients with epilepsy-related tumors. We analyzed the patients’ descriptive statistics, and reported mean, standard deviation (SD), and range (minimum–maximum) for quantitative values. Additionally, the normality assumption of the quantitative data, such as age and duration, was verified. To see the normality assumption, quantile–quantile (Q–Q) and probability–probabilty (P–P) plots were applied. We also used the Shapiro–Wilk test to check the *p*-values for clarity; the result was *p* > 0.05, which indicates that the data follow a normal distribution. We did not apply inferential statistics to see differences between study parameters.

## Discussion

DNTs were reported by Daumas-Duport and Scheithauer first in 1988 and subsequently in another paper published in 1993. They described children with partial complex seizures who were discovered to have cortical brain tumors. Surgical resection of these tumors cured the seizures permanently. On histological examination, these tumors were found to be low grade and morphologically distinct. The authors described the characteristic histological features in detail [15, 16]. In 1993, DNTs was included as a distinct entity in the revised WHO Classification of CNS tumors [17, 18]. A study of 40 cases was published by Daumas-Duport *et al.* in 1999 [19]. These signature studies were followed by a number of case reports and case series that described the clinicopathological features and outcomes of these tumors [20–23]. The most important histologic differential diagnosis of DNT is with oligodendroglioma. DNTs occur in young patients, are associated with a long history of seizures and present as cortical-based expansions without mass effect or edema. These features should suggest the diagnosis of DNT rather than oligodendroglioma. Histologically, they are circumscribed and multinodular rather than diffuse and demonstrate floating neurons. Prominent perineuronal satellitosis is not seen. *IDH* mutation and 1p19q codeletion are not seen [24].

Reiche *et al.* described the computed tomography (CT) and MRI patterns of DNT [22]. Cabiol *et al.* described DNTs as cortical tumors that produced enlargement of a gyrus forming “mega gyrus” that exceeded the normal thickness of the cortex [18]. However, rare case reports have documented extracortical location of DNTs. In such cases, it is important to differentiate DNTs from other extracortical tumors [9]. In a 2003 review, Daumas-Duport and Varlet described DNTs as highly polymorphic tumors that arise preferentially in the supratentorial cortex of young patients with long-term drug-resistant partial seizures, which histologically mimic low-grade gliomas and behave as stable lesions. They noted that DNTs were increasingly being detected by imaging soon after the first seizure and that the seizures were cured by gross total surgical removal of the tumor. They recommended that DNTs should be operated on soon after diagnosis but emphasized that excellent results were also obtained in patients with long-term seizures. They underlined the importance of differentiating DNT from diffuse glioma and sparing these young patients (with normal life expectancy) the long-term harmful effects of unnecessary radiation and chemotherapy [25].

Vaquero *et al.* underscored the importance of considering complex DNT in the differential diagnosis of a cerebellar tumor in a young patient with features of pilocytic astrocytoma. It is important to differentiate DNT from ganglioglioma and other low-grade gliomas since DNTs do not recur following surgery [26]. Pilocytic astrocytomas are typically infratentorial well demarcated cyst-like masses with enhancing mural nodule on imaging and are histologically characterized by a biphasic pattern composed of dense fibrillary areas separated by loosely arranged microcystic areas. They usually demonstrate rosenthal fibers, eosinophilic bodies, and prominent hyalinized blood vessels. Gangliogliomas, like DNTs, are also glioneuronal neoplasms that occur in children and young adults who present with early onset focal epilepsy. Greater than 70% of gangliogliomas are also located in the temporal lobe. On imaging, they appear as intracortical cysts and circumscribed areas of cortical signal enhancement. Histologically, they are composed of dysplastic ganglion cells in combination with neoplastic glial cells producing a biphasic pattern. Microcysts and perivascular lymphocytic infiltrates are often present [1]. DNTs do not usually show the pronounced dysplastic neurons or inflammatory infiltrates typical of gangliogliomas [24].

DNTs usually show typical radiological features presenting as supratentorial masses most commonly located in the temporal lobes without any peritumoral edema or mass effect. However, atypical location and presence of mass effect and peritumoral edema in some cases can make a radiological diagnosis uncertain [27]. Dozza *et al.* in a study of 58 glial and glioneuronal tumors reported that four DNTs had been previously misdiagnosed as astrocytoma (three cases) and oligodendroglioma (one case) [28]. Hoehn *et al.* reported a pontine DNT, which, in addition to the unusual location, presented in a highly atypical manner with acute spontaneous hemorrhage mimicking a cavernous hemangioma [29]. Stark *et al.* reported three DNTs in atypical locations (all three subcortical and bilateral). All three cases had presented with seizures. Thus, DNTs can occur throughout the brain [30]. Sirbu reported a case of sudden death in a 24-year-old woman with a long history of intractable complex partial seizures, which was most likely related to her tumor [31].

There have been occasional case reports documenting aggressive behavior and recurrence in DNT. Takeuchi *et al.* reported a case with rapid regrowth following resection [32]. A number of authors have reported transformation of DNT into a high-grade diffuse glioma [33–36]. The occurrence of recurrence and malignant transformation in some cases legitimizes the long-term surveillance by MRI, especially in cases with subtotal tumor resection [37, 38]. A 2016 study demonstrated increased copy number changes in recurrent DNTs that showed malignant transformation [39].

Over 70% of cases in two series published by Zhang *et al.* and Sakuta *et al.* were complex type [40, 41]. Seven (50%) cases in our series were complex type. Zhang *et al.* reported recurrence in 2 out of 15 cases and suggested that complete resection of the tumor along with the epileptogenic zone was important for a favorable outcome [40]. Long-term recurrence has also been reported. Tonetti *et al.* described a DNT that recurred 24 years after complete surgical resection. The patient remained completely disease-free during this period [42]. Thus, DNTs can recur decades after radiographical complete resection. In children with cortical dysplasia adjacent to the tumor, recurrent intractable seizures can occur emphasizing the importance of complete resection of DNT and all adjacent foci of cortical dysplasia. In cases where adjacent cortical dysplasia is not completely resected, further epilepsy surgery is required. Variable degrees of cortical dysplasia were found in the adjacent brain tissue in majority of cases reported by multiple authors [12, 41, 43]. Studies suggest that the epileptogenic zone is located in the tumor area and cortical reorganization can partly explain functional preservation of cortex following resection [44]. Longer duration of epilepsy before resection has been shown to be associated with worse seizure outcome in patients with DNTs [45–47].

Rare DNTs have been reported in infants [48]. D’Agostino *et al.* described a case of subependymoma and DNT collision tumor [49]. Ravanpay *et al.* reported a case with synchronous occurrence of DNT and oligodendroglioma [50]. Chiang *et al.* described rare seizure-associated midline neoplasms situated near septum pellucidum with histologic features similar to DNT. They proposed that such DNT- like neoplasms of the septum pellucidum (sDNTs) constituted a distinct disease entity, as their genetic alterations were different [51].

At a molecular level, FGFR1 and BRAF alterations, activation and dysregulation of RAS/ERK, P13K/AKT, and mTOR signaling pathways are seen [52, 53]. Internal tandem duplication (ITD) of the tyrosine kinase domain (TKD) of FGFR1is the most prevalent genetic abnormality seen in 40%–60% of cases of DNT, followed by missense mutations in FGFR1. FGFR1 alterations are seen in both sporadic and familial cases. FGFR1 alterations are characteristic but not specific to DNT (TKD duplication is believed to be relatively specific) and are considered the main molecular drivers of DNTs. In addition, according to some studies, BRAF p. V600E mutations are seen in about 50% of DNTs, while they have been found less commonly in others. The wide range may be due to differences in the criteria used for morphologic diagnosis of DNTs in different studies. In fact, some studies did not find BRAF mutations in DNTs containing the specific glioneuronal element. Thus, it is important to include ganglioglioma or MAPK pathway-altered diffuse low-grade glioma in the differential diagnosis when BRAF p. V600E mutation is present. Some groups thus separate epilepsy-associated tumors into those with FGFR1 mutations (DNTs) and those with BRAF mutations. Thus, DNTs are believed to have a distinct methylation and transcriptional profile and most demonstrate FGFR1 mutations. In developing countries, in absence of molecular testing, immunohistochemistry can be utilized for detecting FGFR1 and BRAF molecular alterations [54–59]. PLNTY resembles DNT in being oligodendroglioma-like but has a more infiltrative growth pattern and calcifications. Tumor cells express CD34 and harbor BRAF p. V600E mutations or FGFR2 or FGFR3 fusions. Diffuse astrocytoma, MYB or MYBL1-altered, shows astrocytic cells in a fine bubbly neuropil [60, 61].

Two patients died within days of surgery from surgery-related complications. These cases were received for histopathological examination from centers in other cities. Attempts to contact the surgeons or hospital authorities for additional information/operative notes, and so on, were not successful. Postsurgical complications are a price paid by many patients in smaller cities and towns of developing countries such as Pakistan, due to lack of facilities, infrastructure, and dearth of skilled manpower. The situation is even more critical for brain tumor surgery. In context of epilepsy-related tumors in young patients, the dearth of true epilepsy surgery centers is an important adverse factor. Twinning programs could offer a solution where hospitals/institutions in small cities and towns of the country may collaborate and develop partnerships with academic institutions/hospitals in larger cities to utilize services of expert neurosurgeons in complicated neurosurgery cases.

Seizures continued following surgery in large majority of our patients. However, they were reduced in frequency and severity. All patients were started on tablet Epival 500 mg twice daily, with dose adjustment after checking for valproate levels. In one patient, seizures ceased following resection and the patient is alive and well without seizures and without any antiepileptic treatment.

## Conclusion

In conclusion, correct diagnosis of DNT requires clinicopathological and radiological correlation. MRI is fundamental in differentiating DNTs from other low-grade gliomas. MRI must be performed as soon as possible in young patients suffering from drug-resistant focal epilepsy, and they should undergo early surgical resection if a tumor is discovered. Complete surgical resection of the tumor and adjacent epileptogenic foci and foci of cortical dysplasia is the mainstay of treatment for permanent seizure relief. Recurrences may occur in absence of complete resection of the tumor, and malignant transformation may occur rarely. Since recurrences may occur many years later, long-term follow-up is required. Longer duration of seizures prior to resection may be associated with unsatisfactory seizure control following surgery. Failure to achieve complete tumor resection results in persistence of seizures postsurgery, and patients continue to suffer distressing seizures and need to remain on antiepileptic therapy.

It is important that neurosurgeons, neurooncologists, and pathologists in this part of the world are aware of low-grade epilepsy-related tumors of the young. The importance of a multidisciplinary approach in the diagnosis, surgery, and postsurgical management of these patients cannot be overemphasized. It is also important that a multidisciplinary team, including neurologists, be in place for appropriate management of these patients so that permanent seizure control can be achieved by complete resection, thus enabling these young patients to lead essentially normal lives.

## Data Availability

Data and materials of this work are available from the corresponding author on reasonable request.

## Abbreviations

DNT: Dysembryoplastic neuroepithelial tumor
CNS: Central nervous system
MRI: Magnetic resonance imaging
WHO: World Health Organization
H&E: Hematoxylin and eosin
IHC: Immunohistochemical
ERC: Ethical review committee
GFAP: Glial fibrillary acidic protein
CT: Computed tomography

## References

[1] Pietsch T, Ellison DW, Hirose T, Jacques TS, Schuller U, Varlet P. Dysembryoplastic neuroepithelial tumors. In: 5^th^ edition edited by the WHO Classification of Tumours editorial board, International Agency for Research on Cancer (IARC) Lyon. 2021. pp. 123–6.

[2] Rosemberg S, Vieira GS (1998). Tumor neuroepitelial disembrioplástico. Estudo epidemiológico de uma única instituição. Dysembryoplastic neuroepithelial tumor. An epidemiological study from a single institution. Arq Neuropsiquiatr.

[3] Campos AR, Clusmann H, von Lehe M, Niehusmann P, Becker AJ, Schramm J (2009). Simple and complex dysembryoplastic neuroepithelial tumors (DNT) variants: clinical profile, MRI, and histopathology. Neuroradiology.

[4] Thom M, Toma A, An S, Martinian L, Hadjivassiliou G, Ratilal B (2011). One hundred and one dysembryoplastic neuroepithelial tumors: an adult epilepsy series with immunohistochemical, molecular genetics, and clinical correlations and a review of the literature. J Neuropathol Exp Neurol.

[5] Slegers RJ, Blumcke I (2020). Low-grade developmental and epilepsy associated brain tumors: a critical update 2020. Acta Neuropathol Commun.

[6] Blumcke I, Aronica E, Urbach H, Alexopoulos A, Gonzalez-Martinez JA (2014). A neuropathology-based approach to epilepsy surgery in brain tumors and proposal for a new terminology use for long-term epilepsy-associated brain tumors. Acta Neuropathol.

[7] Raymond AA, Halpin SF, Alsanjari N, Cook MJ, Kitchen ND, Fish DR (1994). Dysembryoplastic neuroepithelial tumor. Features in 16 patients. Brain.

[8] Bourgeois M, Sainte-Rose C, Lellouch-Tubiana A, Malucci C, Brunelle F, Maixner W (1999). Surgery of epilepsy associated with focal lesions in childhood. J Neurosurg.

[9] Cataltepe O, Turanli G, Yalnizoglu D, Topçu M, Akalan N (2005). Surgical management of temporal lobe tumor-related epilepsy in children. J Neurosurg.

[10] Chan CH, Bittar RG, Davis GA, Kalnins RM, Fabinyi GC (2006). Long-term seizure outcome following surgery for dysembryoplastic neuroepithelial tumor. J Neurosurg.

[11] Ishizawa K, Terao S, Kobayashi K, Yoshida K, Hirose T (2007). A neuroepithelial tumor showing combined histological features of dysembryoplastic neuroepithelial tumor and pleomorphic xanthoastrocytoma–a case report and review of the literature. Clin Neuropathol.

[12] Sharma MC, Jain D, Gupta A, Sarkar C, Suri V, Garg A (2009). Dysembryoplastic neuroepithelial tumor: a clinicopathological study of 32 cases. Neurosurg Rev.

[13] Zhang JG, Hu WZ, Li Y, Zhao RJ, Kong LF (2012). Clinicopathologic analysis of dysembryoplastic neuroepithelial tumor. Zhonghua Bing Li Xue Za Zhi.

[14] Suh YL (2015). Dysembryoplastic neuroepithelial tumors. J Pathol Transl Med.

[15] Daumas-Duport C, Scheithauer BW, Chodkiewicz JP, Laws ER, Vedrenne C (1988). Dysembryoplastic neuroepithelial tumor: a surgically curable tumor of young patients with intractable partial seizures. Report of thirty-nine cases. Neurosurgery.

[16] Daumas-Duport C (1993). Dysembryoplastic neuroepithelial tumours. Brain Pathol.

[17] Kleihues P, Burger PC, Scheithauer BW (eds). Histological typing of tumours of the central nervous system. World Health Organization international histological classification of tumours. 2nd ed., Springer-Verlag: Berlin, Heidelberg, New York 1993, p 23.

[18] Cabiol J, Acebes JJ, Isamat F (1999). Dysembryoplastic neuroepithelial tumor. Crit Rev Neurosurg.

[19] Daumas-Duport C, Varlet P, Bacha S, Beuvon F, Cervera-Pierot P, Chodkiewicz JP (1999). Dysembryoplastic neuroepithelial tumors: nonspecific histological forms – a study of 40 cases. J Neurooncol.

[20] Vajtai I, Varga Z, Bodosi M, Kopniczky Z, Kóbor J, Vörös E (1995). Dysembryoplasticus neuroepithelialis tumor [Dysembryoplastic neuroepithelial tumor]. Orv Hetil.

[21] Weissman Z, Michowitz S, Shuper A, Kornreich L, Amir J (1996). Dysembryoplastic neuroepithelial tumor: a curable cause of seizures. Pediatr Hematol Oncol.

[22] Reiche W, Kolles H, Eymann R, Feiden W (1996). Dysembryoplastischer neuroepithelialer Tumor (DNT). Neuroradiologische Befundmuster [Dysembryoplastic neuroepithelial tumor (DNT). Pattern of neuroradiologic findings]. Radiologe.

[23] Terauchi M, Kubota T, Aso T, Maehara T (2006). Dysembryoplastic neuroepithelial tumor in pregnancy. Obstet Gynecol.

[24] Brat DJ, Perry A, Perry A, Brat DJ, Dysembryoplastic Neuroepithelial Tumor (2018). Neuronal and glioneuronal neoplasms. Practical surgical neuropathology. A diagnostic approach.

[25] Daumas-Duport C, Varlet P (2003). Dysembryoplastic neuroepithelial tumors. Rev Neurol.

[26] Vaquero J, Saldaña C, Coca S, Zurita M (2012). Complex form variant of dysembryoplastic neuroepithelial tumor of the cerebellum. Case Rep Pathol.

[27] Guduru H, Shen JK, Lokannavar HS (2012). A rare case of dysembryoplastic neuroepithelial tumor. J Clin Imaging Sci.

[28] Dozza DC, Rodrigues FF, Chimelli L (2012). Dysembryoplastic neuroepithelial tumor originally diagnosed as astrocytoma and oligodendroglioma. Arq Neuropsiquiatr.

[29] Hoehn D, Konoplev S, Yin C (2012). unusual presentation of a Dysembryoplastic neuroepithelial tumors mimiking a pontomesencephalic cavernous Hemangioma. J Med Cases.

[30] Stark J, Friedman E, Thompson S, Von Allmen G, Bhattacharjee M, Tandon N (2018). Atypical presentations of dysembryoplastic neuroepithelial tumors. Epilepsia.

[31] Sîrbu CA (2011). Dysembryoplastic neuroepithelial tumor and probable sudden unexplained death in epilepsy: a case report. J Med Case Rep.

[32] Takeuchi Y, Arakawa Y, Mikami Y, Matsumoto R, Miyamoto S (2014). Dysembryoplastic neuroepithelial tumor with rapid recurrence of pilocytic astrocytoma component. Brain Tumor Pathol.

[33] Aggarwal A, Salunke P, Sodhi HB, Vasishta RK, Gowda KK (2014). Dysembryoplastic neuroepithelial tumor transforming into malignancy: a case report. Neurol India.

[34] Hammond RR, Duggal N, Woulfe JM, Girvin JP (2000). Malignant transformation of a dysembryoplastic neuroepithelial tumor. J Neurosurg.

[35] Sandberg DI, Ragheb J, Dunoyer C, Bhatia S, Olavarria G, Morrison G (2005). Surgical outcomes and seizure control rates after resection of dysembryoplastic neuroepithelial tumors. Neurosurg Focus.

[36] Chao L, Tao XB, Jun YK, Xia HH, Wan WK, Tao QS (2013). Recurrence and histological evolution of dysembryoplastic neuroepithelial tumor: a case report and review of the literature. Oncol Lett.

[37] Garrett M, Eschbacher J, Nakaji P (2008). Dysembryoplastic neuroepithelial tumor: a review. Barrow Quarterly.

[38] Luzzi S, Elia A, Del Maestro M, Elbabaa SK, Carnevale S, Guerrini F (2019). Dysembryoplastic neuroepithelial tumors: what you need to know. World Neurosurg.

[39] Heiland DH, Staszewski O, Hirsch M, Masalha W, Franco P, Grauvogel J (2016). Malignant transformation of a dysembryoplastic neuroepithelial tumor (DNET) characterized by genome-wide methylation analysis. J Neuropathol Exp Neurol.

[40] Zhang JG, Hu WZ, Zhao RJ, Kong LF (2014). Dysembryoplastic neuroepithelial tumor: a clinical, neuroradiological, and pathological study of 15 cases. J Child Neurol.

[41] Sakuta R, Otsubo H, Nolan MA, Weiss SK, Hawkins C, Rutka JT (2005). Recurrent intractable seizures in children with cortical dysplasia adjacent to dysembryoplastic neuroepithelial tumor. J Child Neurol.

[42] Tonetti DA, Ares WJ, Richardson RM, Hamilton RL, Lieberman FS (2017). Long-term recurrence of dysembryoplastic neuroepithelial tumor: clinical case report. Surg Neurol Int.

[43] Chen L, Xu QZ, Piao YS, Zhang GJ, Yu T, Yang XP, Yang H, Lu DH (2007). Dysembryoplastic neuroepithelial tumor: a clinicopathologic and immunohistochemical study. Zhonghua Bing Li Xue Za Zhi.

[44] Xue H, Sveinsson O, Li YJ (2015). Resection of a dysembryoplastic neuroepithelial tumor in the precentral gyrus. World J Pediatr.

[45] Isler C, Erturk Cetin O, Ugurlar D, Ozkara C, Comunoglu N, Kizilkilic O (2018). Dysembryoplastic neuroepithelial tumours: clinical, radiological, pathological features and outcome. Br J Neurosurg.

[46] Nolan MA, Sakuta R, Chuang N, Otsubo H, Rutka JT, Snead OC (2004). Dysembryoplastic neuroepithelial tumors in childhood: long-term outcome and prognostic features. Neurology.

[47] Aronica E, Leenstra S, van Veelen CW, van Rijen PC, Hulsebos TJ, Tersmette AC, Yankaya B, Troost D (2001). Glioneuronal tumors and medically intractable epilepsy: a clinical study with long-term follow-up of seizure outcome after surgery. Epilepsy Res.

[48] Wang H, Ye JT, Yao HX, Li D, Dong Y (2017). Clinicopathologic features of infant dysembryoplastic neuroepithelial tumor: a case report and literature review. Beijing Da Xue Xue Bao Yi Xue Ban.

[49] D'Agostino E, Calnan DR, Hickey W, Bauer DF (2019). Subependymoma and dysembryoplastic neuroepithelial collision tumor in the foramen of Monro: case report. J Neurosurg Pediatr.

[50] Ravanpay AC, Gabikian P, Marshall D, Williams JR, Huber B, Silbergeld DL (2019). Synchronous identification of a dysembryoplastic neuroepithelial tumor (DNET) and an oligodendroglioma in a patient: a case report. Clin Neuropathol.

[51] Chiang JCH, Harreld JH, Tanaka R, Li X, Wen J, Zhang C (2019). Septal dysembryoplastic neuroepithelial tumor: a comprehensive clinical, imaging, histopathologic, and molecular analysis. Neuro Oncol.

[52] Sontowska I, Matyja E, Malejczyk J, Grajkowska W (2017). Dysembryoplastic neuroepithelial tumour: insight into the pathology and pathogenesis. Folia Neuropathol.

[53] Surrey LF, Jain P, Zhang B, Straka J, Zhao X, Harding BN (2019). Genomic analysis of dysembryoplastic neuroepithelial tumor spectrum reveals a diversity of molecular alterations dysregulating the MAPK and PI3K/mTOR pathways. J Neuropathol Exp Neurol.

[54] Blümcke I, Aronica E, Becker A, Capper D, Coras R, Honavar M, Jacques TS, Kobow K, Miyata H, Mühlebner A, Pimentel J (2016). Low-grade epilepsy-associated neuroepithelial tumours—the 2016 WHO classification. Nat Rev Neurol.

[55] Blümcke I, Coras R, Wefers AK, Capper D, Aronica E, Becker A, Honavar M, Stone TJ, Jacques TS, Miyata H, Mühlebner A (2019). Challenges in the histopathological classification of ganglioglioma and DNT: microscopic agreement studies and a preliminary genotype-phenotype analysis. Neuropathol Appl Neurobiol.

[56] Fina F, Barets D, Colin C, Bouvier C, Padovani L, Nanni-Metellus I, Ouafik LH, Scavarda D, Korshunov A, Jones DT, Figarella-Branger D (2017). Droplet digital PCR is a powerful technique to demonstrate frequent FGFR1 duplication in dysembryoplastic neuroepithelial tumors. Oncotarget.

[57] Matsumura N, Nobusawa S, Ito J, Kakita A, Suzuki H, Fujii Y, Fukuda M, Iwasaki M, Nakasato N, Tominaga T, Natsume A (2019). Multiplex ligation-dependent probe amplification analysis is useful for detecting a copy number gain of the FGFR1 tyrosine kinase domain in dysembryoplastic neuroepithelial tumors. J Neurooncol.

[58] Rivera B, Gayden T, Carrot-Zhang J, Nadaf J, Boshari T, Faury D, Zeinieh M, Blanc R, Burk DL, Fahiminiya S, Bareke E (2016). Germline and somatic FGFR1 abnormalities in dysembryoplastic neuroepithelial tumors. Acta Neuropathol.

[59] Qaddoumi I, Orisme W, Wen J, Santiago T, Gupta K, Dalton JD, Tang B, Haupfear K, Punchihewa C, Easton J, Mulder H (2016). Genetic alterations in uncommon low-grade neuroepithelial tumors: BRAF, FGFR1, and MYB mutations occur at high frequency and align with morphology. Acta Neuropathol.

[60] Huse JT, Snuderl M, Jones DT, Brathwaite CD, Altman N, Lavi E, Saffery R, Sexton-Oates A, Blumcke I, Capper D, Karajannis MA (2017). Polymorphous low-grade neuroepithelial tumor of the young (PLNTY): an epileptogenic neoplasm with oligodendroglioma-like components, aberrant CD34 expression, and genetic alterations involving the MAP kinase pathway. Acta Neuropathol.

[61] Wefers AK, Stichel D, Schrimpf D, Coras R, Pages M, Tauziède-Espariat A, Varlet P, Schwarz D, Söylemezoglu F, Pohl U, Pimentel J (2020). Isomorphic diffuse glioma is a morphologically and molecularly distinct tumour entity with recurrent gene fusions of MYBL1 or MYB and a benign disease course. Acta Neuropathol.

